# HIF-1α Mediated Regulation of Glioblastoma Malignant Phenotypes through CD47 Protein: Understanding Functions and Mechanisms

**DOI:** 10.7150/jca.101050

**Published:** 2025-01-01

**Authors:** Qijia Tan, Feng Li, Jun Wang, Yuxi Zou, Yanping Tang, Yingqian Cai, Xiaodan Jiang

**Affiliations:** 1 Neurosurgery Center, The National Key Clinical Specialty, The Engineering Technology Research Center of Education Ministry of China on Diagnosis and Treatment of Cerebrovascular Disease, Guangdong Provincial Key Laboratory on Brain Function Repair and Regeneration, The Neurosurgery Institute of Guangdong Province, Zhujiang Hospital, Southern Medical University, Guangzhou 510282, China.; 2 Department of Neurosurgery, Guangdong Provincial Hospital of Chinese Medicine, The Second Affiliated Hospital of Guangzhou University of Chinese Medicine, Guangzhou 510120, China.

**Keywords:** glioblastoma, HIF-1α, CD47, proliferation, migration

## Abstract

Glioblastoma (GBM) is a highly invasive and malignant primary intracranial tumor originating from glial cells, and it is associated with an extremely poor clinical prognosis. The hypoxic conditions within GBM promote various tumor cell processes such as angiogenesis, proliferation, migration, invasion, and drug resistance. A key aspect of tumor adaptation to the hypoxic environment and the promotion of malignant behaviors is the regulation of HIF-1α signaling pathways. However, the specific pathogenic mechanisms involving HIF-1α in GBM have not been thoroughly investigated. This study reveals significant overexpression of both HIF-1α and CD47 in GBM. Patients with high HIF-1α levels and CD47 expression had significantly reduced overall survival and disease-free survival times. Furthermore, a positive correlation was observed between the expression levels of HIF-1α and CD47 in GBM. Lentivirus-mediated knockdown of HIF-1α protein and plasmid-based overexpression of CD47 protein simultaneously enhanced cell proliferation, clonogenic potential and cell migration abilities in GBM, and HIF-1α was found to regulate key pathways, including the P-PI3K/P-AKT, SOX2/OCT4 and MMP2/MMP9 pathways, in GBM.

## Introduction

Glioblastoma Multiforme (GBM) is a highly aggressive and malignant brain tumor that poses significant challenges in diagnosis, treatment, and prognosis evaluation. Despite recent advancements, the 5-year survival rate of GBM patients remains dismal^1^, ^2^. HIF-1α protein, a transcription factor activated in response to hypoxic conditions, has emerged as a pivotal regulator in various biological processes, including cell proliferation, apoptosis, angiogenesis, and metabolism^3^-^5^. Recent studies have underscored the critical role of HIF-1α in the genesis and progression of GBM. Notably, the expression level of HIF-1α protein in GBM significantly surpasses that in normal brain tissue, closely correlating with the tumor's malignant behavior. HIF-1α fosters the upregulation of genes involved in tumor proliferation, invasion, and angiogenesis, thereby augmenting GBM growth and metastasis. Furthermore, HIF-1α assumes a pivotal role in stimulating the proliferation and invasion of GBM cells by elevating the expression of Cyclin D1, C-MYC, and VEGF^6^, ^7^. Additionally, HIF-1α exerts inhibitory effects on apoptosis and autophagy in GBM cells, further amplifying their proliferative and invasive abilities^8^. Thus, targeting HIF-1α has become a promising strategy for GBM treatment.

CD47 protein is significantly overexpressed in GBM compared to normal brain tissues. Extensive studies have revealed a strong correlation between high CD47 expression and the malignant behavior characteristic of GBM. CD47 functions by inhibiting macrophage phagocytosis of tumor cells through its binding to signal regulatory protein α (SIRPα) on the surface of macrophages, thereby promoting tumor growth and metastasis^9^-^11^. Additionally, CD47 promotes the proliferation, invasion, and angiogenesis of tumor cells, further driving the malignant progression of GBM, including the upregulation of key factors such as Cyclin D1, c-Myc, and MMP9^12^, ^13^. Based on these findings, CD47 is considered one of the important targets for GBM treatment.

Research has found that CD31 primarily regulates the adhesion of endothelial cells in the tumor microenvironment (TME) and promotes tumor cell proliferation, rather than stimulating tumor angiogenesis. When CD31 is expressed on tumor cells, it transmits signals to activate multiple pathways that mediate anti-tumor cell apoptosis. In hepatocellular carcinoma cells, studies have confirmed the expression of CD31 on MHCC97H and HCC-LM3 lines, and verified that CD31 induces EMT by upregulating integrin β1, thereby activating the FAK-Akt pathway. Additionally, related research has discovered that in GBM, HIF-1α regulates the expression level of CD31 to promote tumor progression^11^, ^13^.

The PI3K/AKT pathway is a pivotal signaling cascade associated with the regulation of essential cellular processes, including cell growth, survival and metabolism, and its dysregulation has been implicated in numerous human diseases, including cancer^14^. Notably, the PI3K/AKT pathway has been shown to undergo aberrant activation in several cancer types, including GBM, which is the most prevalent primary brain tumor in adults. In GBM, the PI3K/AKT pathway plays a critical role in the survival and proliferation of Cancer Stem Cells (CSCs)^15^, ^16^, and its activation leads to the phosphorylation and activation of several downstream targets, including mTOR, GSK3β and FOXO3a, which can regulate cell growth, metabolism, and survival. In GBM, the PI3K/AKT pathway is often constitutively activated due to various genetic and epigenetic alterations, such as mutations in PI3KCA, PTEN and AKT1/2/3 genes, as well as loss of PTEN expression and activation of oncogenic receptor tyrosine kinases (RTKs) such as PDGFRA and IDH1R132H^17^-^19^.

SOX2 and OCT4 are crucial transcription factors typically associated with embryonic and pluripotent stem cells, regulating essential cellular processes like proliferation, differentiation and self-renewal. In recent years, an increasing number of studies have highlighted the pivotal role of the SOX2/OCT4 signaling pathway in the development and progression of glioma^16^, ^20^. SOX2, characterized by its HMG-box domain, functions by binding to DNA and controlling the expression of downstream genes, while OCT4, with its POU domain, collaborates with SOX2 to jointly regulate the expression of these downstream genes. Elevated SOX2 and OCT4 expression in GBM cells has been shown to stimulate glioma cell proliferation and invasion through multiple mechanisms^21^, ^22^. For instance, SOX2 can upregulate the expression of genes such as Cyclin D1 and c-Myc to promote glioma cell proliferation. Conversely, OCT4 can increase the expression of genes such as Nanog and Sox2 to facilitate glioma cell self-renewal and invasion. Moreover, the SOX2/OCT4 signaling pathway can suppress the apoptosis of glioma cells by inhibiting the activity of pro-apoptotic factors (i.e., BAD and CASPASE), thereby further enhancing the malignant progression of glioma cells^23^.

The matrix metalloproteinases (MMPs) family comprises a group of enzymes that are pivotal for the degradation and restructuring of the extracellular matrix (ECM), which offers structural support and governs cellular behavior. This family contains various members, including collagenases, gelatinases and membrane-type MMPs, each playing crucial roles in diverse biological processes such as embryonic development, tissue repair, inflammation and cancer progression^24^. Recent investigations have linked the MMPs family to the pathogenesis of GBM, which is characterized by abnormal glial cell proliferation, irregular blood vessel formation and extensive necrosis. It has also been suggested that the MMPs family actively participates in GBM biology and could be potential therapeutic targets. For instance, the knockdown of MMP-2 overexpression in GBM tissues and cell lines inhibited GBM cell proliferation and invasion both *in vitro* and *in vivo*^25^. Similarly, elevated expression of MMP-9 has been observed in GBM tissues compared to normal brain tissues. The use of small molecule inhibitors or shRNA to inhibit MMP-9 has been demonstrated to reduce GBM cell viability and migration. Furthermore, members of the MMPs family have been implicated in various other aspects of GBM biology^26^. For instance, MMP-9 has been found to regulate the activity of several signaling pathways essential for GBM growth and survival, including the PI3K/AKT/mTOR, Wnt/β-catenin and Notch pathways, and its knockdown or inhibition has been shown to suppress these pathways, resulting in reduced GBM cell viability and tumor growth, both *in vitro* and *in vivo*^27^.

In this study, we observe increased expression of HIF-1α and CD47 in GBM. Knocking down the HIF-1α protein significantly reduced the malignant proliferation, clone-forming and migration abilities of GBM cells. Conversely, the simultaneous knockdown of HIF-1α protein using lentivirus and overexpression of CD47 protein via plasmid increased cell proliferation, clone forming and migration abilities in GBM, indicating that HIF-1α may influence GBM function through CD47. *In vivo* experiments demonstrate that suppressing HIF-1α protein expression reduces the growth rate of subcutaneously formed tumors and decreases the expression levels of proteins associated with the P-PI3K/P-AKT, SOX2/OCT4, and MMP2/MMP9 pathways within the tumor. Overall, this study reports that HIF-1α contributes to GBM tumorigenesis by modulating the activation of the P-PI3K/P-AKT, SOX2/OCT4 and MMP2/MMP9 pathway proteins through its interaction with CD47 protein.

## Materials and Methods

### Cell culture

Human astrocytoma cell line HA1800 and human glioblastoma cell lines U87 and U251 were cultured at 37°C in an atmosphere containing 5% CO_2_ in Dulbecco's modified Eagle's medium (DMEM) supplemented with 10% Fetal Bovine Serum (Gibco, Carlsbad, CA, USA) and 1% penicillin/streptomycin (Gibco). To create hypoxic conditions, 5 × 10^4^ cells per well were incubated in an environment with 1% oxygen for 24 hours using 6-well plates (Costar).

### Western blotting

Western blotting was conducted following a standard protocol. Total proteins were extracted using radio-immunoprecipitation assay (RIPA) buffer containing 1x phosphatase inhibitor and proteinase inhibitor (Beyotime Biotechnology, Shanghai, China). The proteins were then separated by SDS-PAGE and transferred onto PVDF membranes (Millipore, Billerica, MA) using electroblotting. To prevent the non-specific binding of proteins to the membrane, the PVDF membranes were initially blocked with either 5% non-fat dried milk or BSA. This step was crucial to ensure that only the targeted antibodies would bind to their specific protein targets. Following the blocking step, the membranes were incubated with monoclonal antibodies specific to the proteins of interest. These antibodies formed complexes with their respective target proteins on the membrane, which could be detected in subsequent analysis. To visualize the binding of antibodies to the membrane, an ECL luminol solution (Thermo Fisher Scientific, Waltham, MA) was applied. Finally, an image quantification instrument (Chemi-DocTH-XRS+) was used to quantify the bands on the membrane.

### RT-qPCR

Total RNA was extracted using Trizol reagent (Takara, Japan) following the manufacturer's instructions. Subsequently, first-strand cDNA was synthesized from 1 μg of total RNA using random primers (Takara, Japan). Quantitative RT-PCR was performed using primer sets obtained from Sangon (Shanghai, China). The relative mRNA levels were normalized to Actin expression, and the results were calculated using the ΔΔCt method. The primer sequences are provided in Table 1.

### Cell transfection

ShRNA targeting HIF-1α was synthesized by Genechem (Shanghai, China), and the expression vector encoding human CD47 genes was obtained from Sangon (Shanghai, China). A total of 5.0 μL of 2.4 x 10^9^ shRNA molecules were introduced into the cells. Transfected cells were used for functional assays after 7 days. For the transfection of CD47 expression vectors, 2 µg of plasmids were transfected into GBM cells using Lipo2000 (Invitrogen, Carlsbad, CA, USA) following the manufacturer's instructions. Cells transfected with an empty vector served as negative controls. The sequences of shRNA are provided in Table 2.

### Cell proliferation using CCK8 assay

We first seeded approximately 5×10^3^ GBM cells in each well of a 96-well plate and treated the cells with shRNA or plasmids and the corresponding negative controls. After 12, 24, 36 and 48 hours of incubation, the Cell Counting Kit-8 (CCK-8) assay (Beyotime Biotechnology, Shanghai, China) was used to measure the cell viability according to the manufacturer's protocol. To perform the CCK-8 assay, we first added 10 μL of CCK-8 solution to each well of the 96-well plate and incubated the plates for 2 hours at 37°C in a humidified atmosphere with 5% CO_2_. We then measured the absorbance at 450 nm using a microplate reader.

### Cell clonogenic assay

For the clonogenic assay, we seeded 500 cells per well in 6-well plates and subjected them to 1% oxygen conditions for 24 hours. We monitored the cells for 7 days until visible colonies had formed. Once the colonies were visible, we fixed them with methanol for 15 minutes and subsequently stained them with crystal violet for 5 minutes. Finally, we employed an optical system to capture images and conduct statistical analysis.

### Cell migration assay

In the transwell migration assay, we seeded 2 × 10^5^ cells onto the upper chamber containing 0.5 ml of serum-free medium. The lower chamber was filled with complete medium containing 10% FBS, and the cells were incubated for 24 hours. Afterward, we removed the non-migrating cells from the upper chambers, fixed the migratory cells in 4% formaldehyde in PBS at room temperature for 20 minutes, and stained them with crystal violet. The number of migrated cells was quantified as the average count from 5 randomly selected microscopic fields.

### Subcutaneous tumorigenesis assay

We conducted subcutaneous tumorigenesis assays using 4-6-week-old female BALB/c nude mice. Each mouse received a subcutaneous implantation of 5×10^6^ U87 cells stably expressing shRNA-HIF-1α, and we monitored tumor formation for approximately one month. Throughout the study, we measured both tumor growth and body weight every 5 days. To determine tumor size, we used a vernier caliper to measure the length and width, and then calculated tumor volume using the formula: Tumor volume = 1/2 (length × width^2^). All animal procedures were performed in accordance with the guidelines and regulations approved by the Institutional Animal Care and Use Committee.

### Kindey and liver function detection

The AST, ALT and BUN levels in the serum were determined using enzyme-linked immunosorbent assay (ELISA) kits (Shanghai Institute of Biological Products Co., Ltd., Shanghai, China).

### GEPIA database analysis

GEPIA, as an online database for gene expression and prognosis analysis, offers a user-friendly interface. To utilize this resource, users can open a web browser and visit the official GEPIA website. On the homepage, an input field allows entry of the gene's name or symbol for analysis, and the analysis process is initiated by clicking the "Search" button, granting access to the gene expression and prognosis analysis section. In this section, users can explore the gene's expression patterns across various tissues. By default, the data is displayed as a heatmap, with darker shades representing higher levels of gene expression. Additionally, the database provides insights into the gene's expression levels in both normal and cancerous tissues. GEPIA facilitates survival analysis, enabling investigations into potential correlations between gene expression and patients' survival durations. In the gene expression profile segment, researchers can specify the relevant sample type, clinical parameters, and data types. Subsequently, the preferred survival analysis method, such as univariate or multivariate survival analysis, can be selected to create survival curves and obtain the corresponding statistical test outcomes.

### Statistical analysis

The data were analyzed using GraphPad Prism software, and the results are presented as mean ± standard deviation (SD). Statistical significance was assessed using an unpaired two-tailed Student's t-test. Grayscale values were analyzed using Image-J software. A significance level of *P* < 0.05 was considered to indicate statistical significance.

## Results

### HIF-1α is highly expressed in GBM and its expression significantly increases under hypoxic conditions

**Figure 1:**
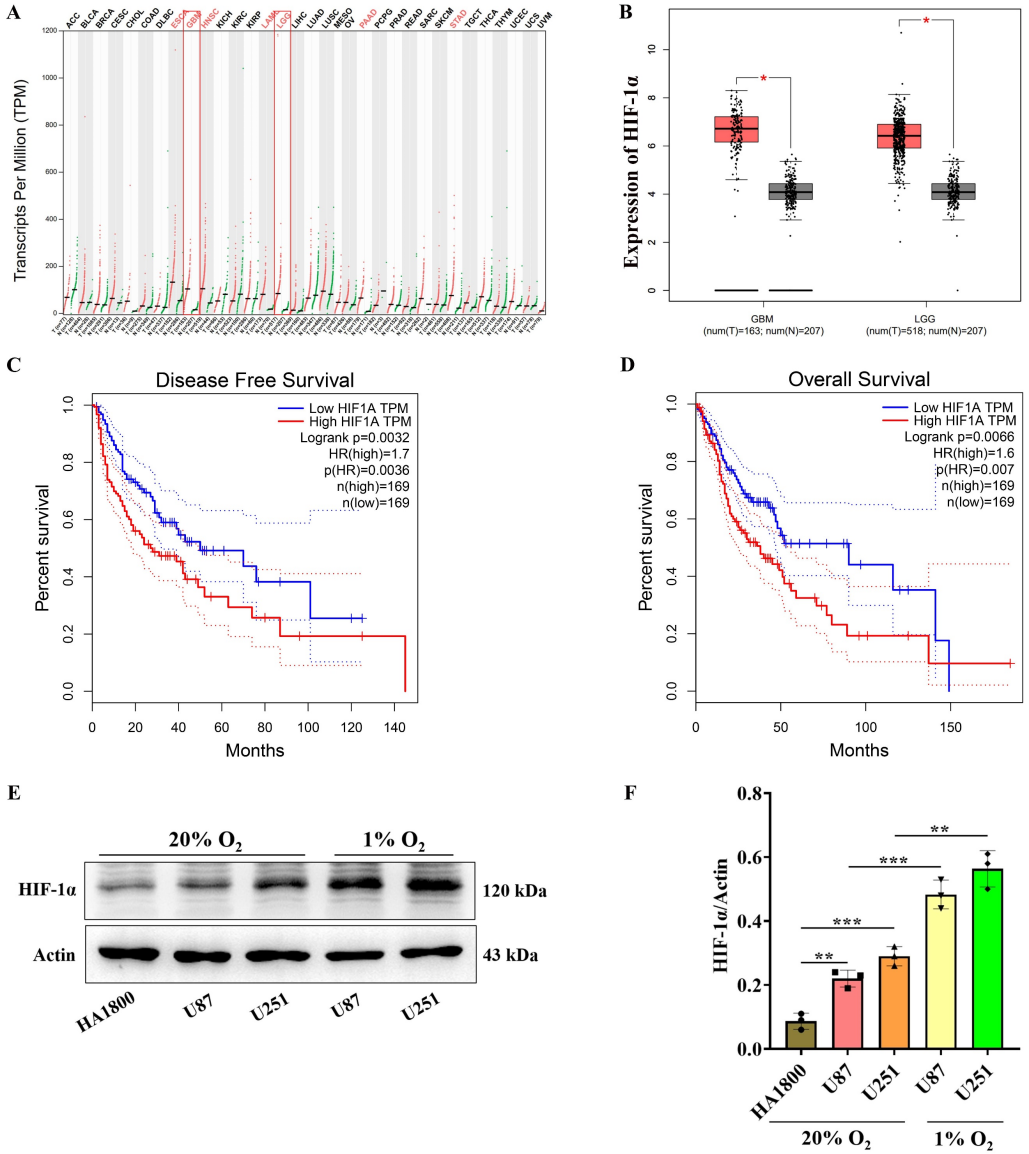
** HIF-1α is highly expressed in GBM.** (A) Expression levels of HIF-1α gene across various tumor types; (B) Expression levels of HIF-1α gene in low-grade and high-grade gliomas. (C) Analysis of disease-free survival time in patients with varying HIF-1α gene expression levels; (D) Analysis of overall survival time in patients with varying HIF-1α gene expression levels. (E) Western blot analysis showing HIF-1α protein expression levels in glioblastoma; (F) Graphical representation of statistical analysis for HIF-1α protein expression levels. Data are presented as mean ± standard deviation, and unpaired t-test was used to compare differences between two groups. *P <0.05, **P <0.01, ***P <0.001.

Gene Expression Profiling Interactive Analysis (GEPIA) is an online resource facilitating gene expression research and survival analysis^28^. Herein, the GEPIA database was used to visualize the expression level of the HIF-1α gene in multiple tumors, which revealed significant overexpression of HIF-1α in ESCA, GBM, HNSC, LAML, LGG, PAAD, STAD and other tumors compared to normal controls (Figure 1A). Additionally, we utilized GEPIA to visualize HIF-1α expression levels in both low-grade glioma and high-grade glioma, establishing that HIF-1α exhibited overexpression in glioma relative to normal controls (Figure 1B). Survival analysis using GEPIA based on 169 patients with HIF-1α overexpression and 169 patients with HIF-1α under-expression showed that the disease-free survival time of patients with HIF-1α overexpression was significantly lower compared to patients with HIF-1α under-expression (Figure 1C). Similarly, patients with HIF-1α overexpression had significantly reduced overall survival compared to patients with HIF-1α under-expression (Figure 1D). These findings underscore HIF-1α as a prominent high-risk factor influencing the prognosis of glioma patients.

Next, we conducted immunoblotting experiments to assess HIF-1α gene expression in glioblastoma *in vitro*. HIF-1α is known to be expressed in various tumors, particularly in conditions characterized by hypoxia. In the context of tumor growth and metastasis, hypoxia frequently occurs due to inadequate angiogenesis and increased tumor cell metabolism, under which HIF-1α becomes activated, facilitating tumor cell adaptation and survival^4^. To investigate this further, we cultured glioblastoma cells U87 and U251 under both normal (20% O_2_) and hypoxic (1% O_2_) conditions for 24 hours and found that under normal culture conditions, the expression levels of HIF-1α protein in U87 and U251 cells exceeded those observed in the control cell line HA1800. Furthermore, in hypoxic culture conditions, the expression levels of HIF-1α protein were significantly elevated compared to those observed under normal culture conditions in both U87 and U251 cells (Figure 1E-F). Collectively, as these findings strongly indicate the overexpression of HIF-1αin glioblastoma, we further investigate the mechanisms through which HIF-1α protein overexpression contributes to its malignancy.

### HIF-1α promotes the malignant proliferation, clonal formation, and migration functions of GBM

**Figure 2:**
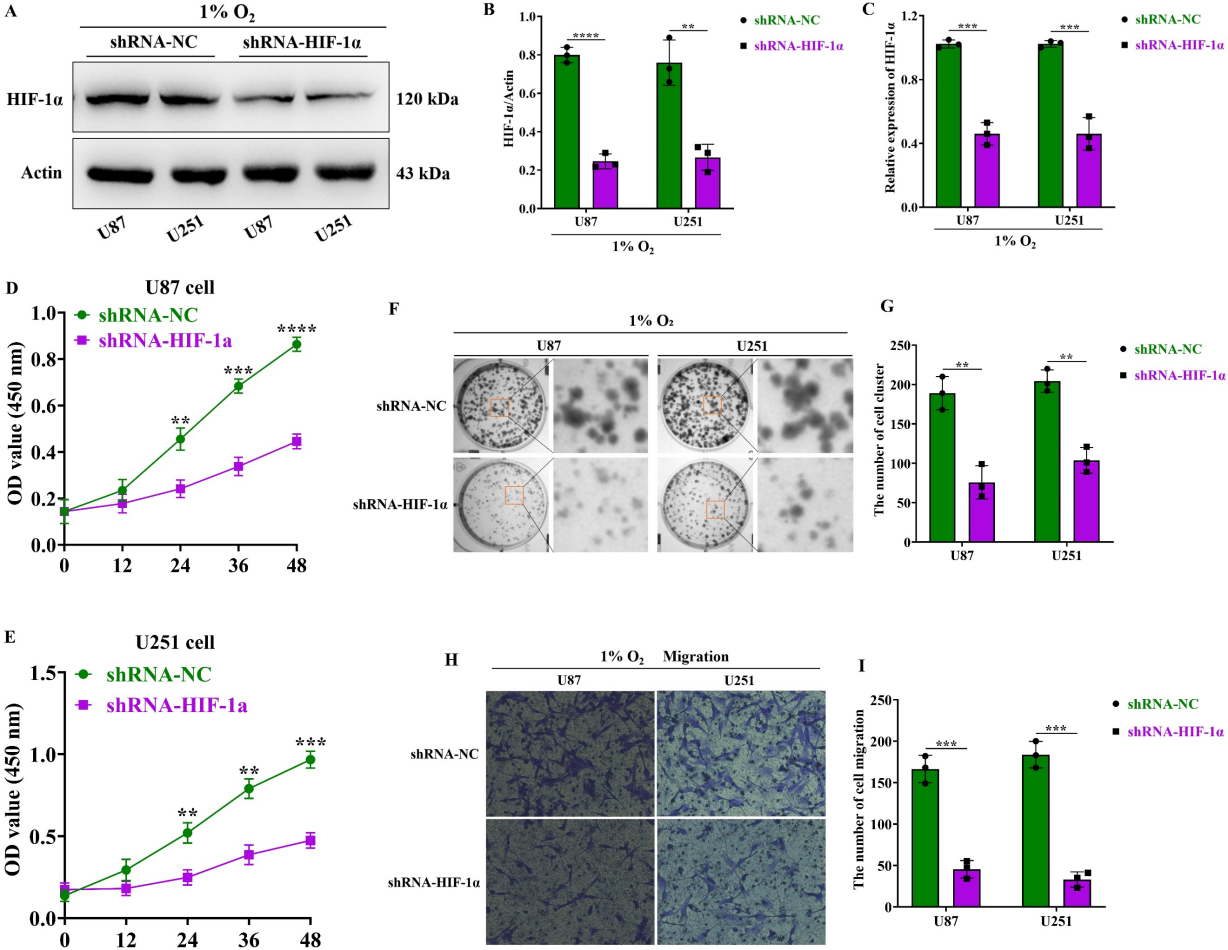
**HIF-1α promotes the malignant progression of GBM.** (A) Western blot analysis of HIF-1α protein expression levels in GBM; (B) Graphical representation of HIF-1α protein expression levels; (C) Real-time quantitative PCR assessing HIF-1α mRNA expression in GBM. (D) CCK8 assay to measure U87 cell proliferation following HIF-1α knockdown; (E) CCK8 assay to measure U251 cell proliferation following HIF-1α knockdown. (F) Clonogenic assay to assess the colony-forming ability of U87 and U251 cells after HIF-1α knockdown; (G) Statistical analysis of the number of colonies formed. (H) Cell migration assay to evaluate U87 and U251 cell migration post-HIF-1α knockdown; (I) Statistical analysis of migrated cell numbers. Data are presented as mean ± standard deviation, and differences between two groups were analyzed using an unpaired t-test. **P <0.01, ***P <0.001, ****P <0.0001.

To further investigate the function of HIF-1α in GBM, we knocked down HIF-1α expression using lentivirus. Real-time fluorescence quantitative PCR (RT-qPCR) experiments revealed a significant reduction in HIF-1α mRNA levels in both U87 and U251 cells within the HIF-1α lentivirus knockdown group (shRNA-HIF-1α) compared to the lentivirus control group (shRNA-NC) (Figure 2C). Additionally, under hypoxic conditions, immunoblotting experiments demonstrated significantly lower HIF-1α protein expression levels in the shRNA-HIF-1α group for both U87 and U251 cells compared to the shRNA-NC group (Figure 2A-B). Overall, these results validate the successful knockdown of HIF-1α protein expression in GBM cells using lentivirus.

To investigate the function of HIF-1α protein in GBM, we first assessed the proliferation of GBM cells using the CCK8 assay and found that under hypoxic conditions, the proliferation capacity of U87 and U251 cells in the shRNA-HIF-1α group began to decline after 24 hours of culture, and with extended culture periods (36-48 hours), their proliferation ability was significantly reduced compared to the shRNA-NC group (Figure 2D-E). Furthermore, we examined GBM colony formation via cell colony formation assay, which demonstrated that under hypoxic conditions, the colony formation of U87 and U251 cells in the shRNA-HIF-1α group was markedly reduced compared to the shRNA-NC group (Figure 2F-G). Additionally, our cell migration assay revealed that under hypoxic conditions, the migration ability of U87 and U251 cells in the shRNA-HIF-1α group was significantly impaired compared to the shRNA-NC group (Figure 2H-I). Collectively, these findings provide evidence that elevated HIF-1α protein expression promotes various malignant traits in GBM.

### HIF-1α activates the P-PI3K/P-AKT, SOX2/OCT4 and MMP2/MMP9 signaling pathways in GBM

**Figure 3:**
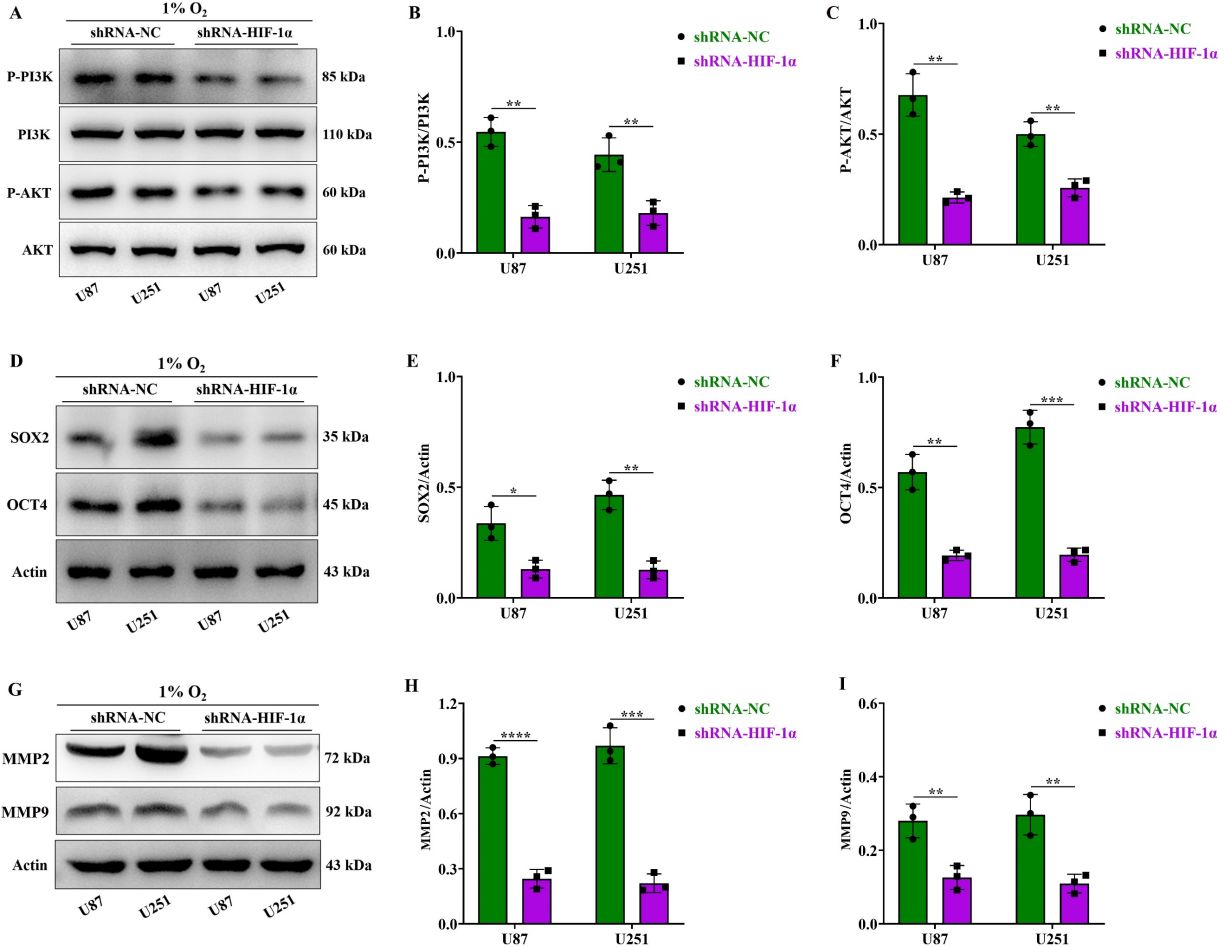
** HIF-1α activates the P-PI3K/P-AKT, SOX2/OCT4 and MMP2/MMP9 signaling pathways in GBM.** (A) Western blot analysis of P-PI3K, PI3K, P-AKT, and AKT protein expression in GBM; (B) Expression levels of P-PI3K and PI3K proteins graphed; (C) Expression levels of P-AKT and AKT proteins graphed; (D) Western blot analysis of SOX2 and OCT4 protein expression in GBM; (E) Expression levels of SOX2 protein graphed; (F) Expression levels of OCT4 protein graphed; (G) Western blot analysis of MMP2 and MMP9 protein expression in GBM; (H) Expression levels of MMP2 protein graphed; (I) Expression levels of MMP9 protein graphed. Data are presented as mean ± standard deviation, and differences between two groups were analyzed using an unpaired t-test. *P <0.05, **P <0.01, ***P <0.001, ****P <0.0001.

The P-PI3K/P-AKT pathway is recognized for its role in promoting tumor cell growth and proliferation^29^. Our experiments conducted under hypoxic conditions showed decreased expression levels of P-PI3K/P-AKT proteins in U87 and U251 cells within the shRNA-HIF-1α group compared to the shRNA-NC group. Importantly, there was no significant difference in the expression levels of total PI3K and AKT proteins (Figure 3A-C). Next, we investigated the changes in proteins related to the cell clonal signaling pathway. SOX2 and OCT4, two pivotal stem cell transcription factors, exert significant influence on tumor initiation and progression^30^. Our experimental findings under hypoxic conditions indicated that the shRNA-HIF-1α group exhibited reduced expression levels of SOX2 and OCT4 proteins in U87 and U251 cells when compared to the shRNA-NC group (Figure 3D-F). We further investigated protein alterations in the cell migration signaling pathway, particularly focusing on the MMPs pathway, known for its pivotal role in tumor migration and is instrumental in degrading the extracellular matrix (ECM), a critical step in tumor invasion and metastasis^31^. Our findings showed that under hypoxic conditions, the expression levels of MMP2 and MMP9 proteins in the U87 and U251 cells decreased in the shRNA-HIF-1α group (Figure 3G-I), suggesting that HIF-1α can regulate multiple signaling pathways to promote the malignant progression of GBM.

### HIF-1α is positively correlated with CD47 expression and promotes the high expression of CD47 in GBM

**Figure 4:**
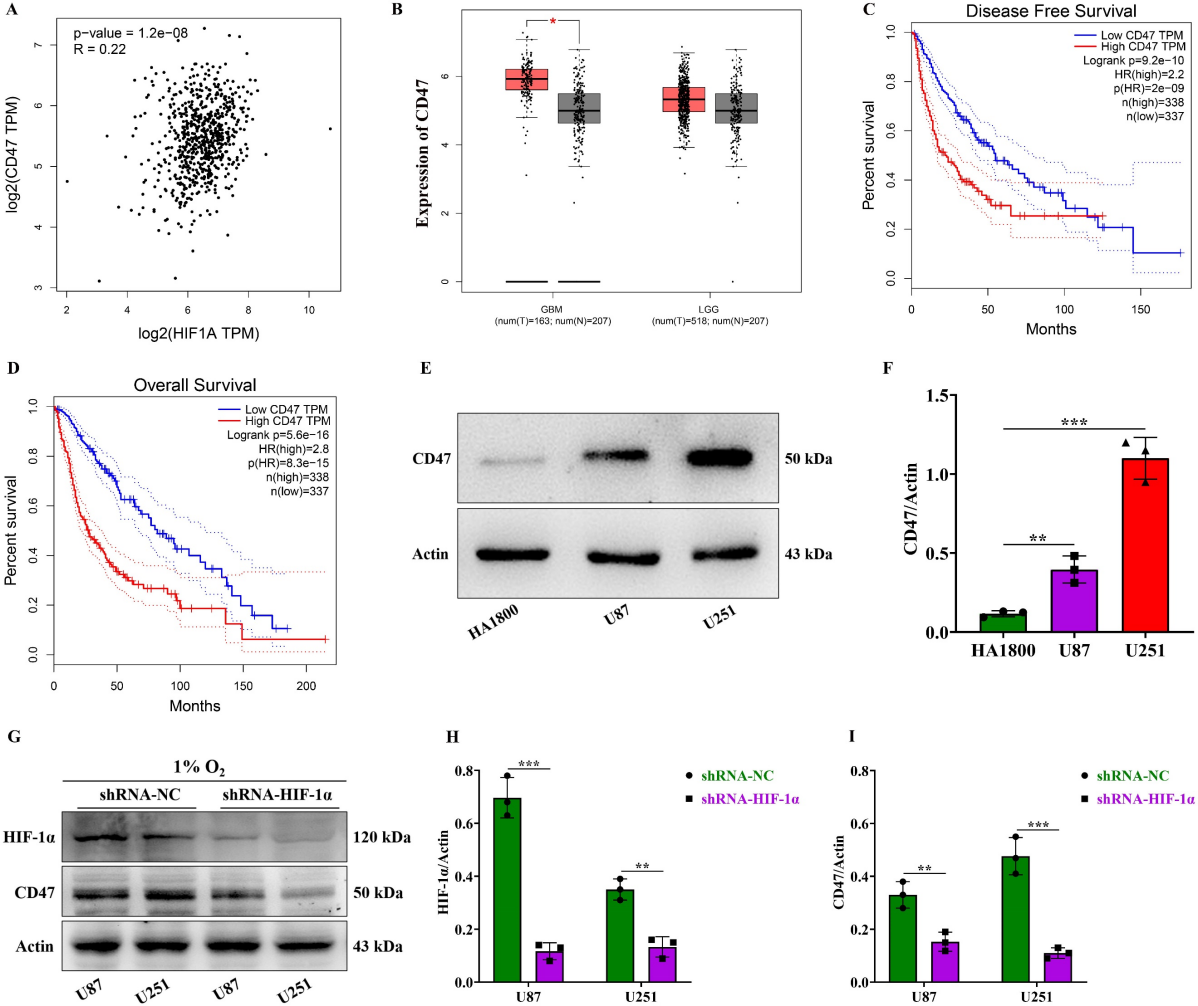
** HIF-1α is positively correlated with CD47 expression and promotes the high expression of CD47 in GBM.** (A) Pearson correlation analysis between HIF-1α and CD47 gene expression levels; (B) GEPIA database visualization of CD47 gene expression in gliomas; (C) Disease-free survival analysis for patients with differing CD47 gene expression levels; (D) Overall survival analysis for patients with differing CD47 gene expression levels; (E) Western blot of CD47 protein expression in GBM; (F) CD47 protein expression levels graphed; (G) Western blot of HIF-1α and CD47 proteins in GBM; (H) HIF-1α protein expression levels graphed; (I) CD47 protein expression levels graphed. Data are shown as mean ± standard deviation, and differences were assessed with an unpaired t-test. **P <0.01, ***P <0.001.

To identify target genes regulated by HIF-1α, we analyzed HIF-1α's correlated gene expression in the GEPIA database. Our analysis revealed a positive correlation between HIF-1α and CD47 gene expression in gliomas, with an associated correlation coefficient of 0.22 (Figure 4A). This suggests that HIF-1α may contribute to glioma's malignant progression by upregulating CD47 expression. Subsequently, we examined CD47 gene expression in both low-grade and high-grade gliomas using the GEPIA database. While no statistically significant difference was observed in CD47 gene expression between low-grade gliomas, high-grade gliomas exhibited higher CD47 gene expression compared to normal controls (Figure 4B). Furthermore, our analysis demonstrated that patients with high CD47 gene expression experienced significantly shorter disease-free survival compared to those with low CD47 gene expression (Figure 4C). Similarly, patients with high CD47 gene expression exhibited notably reduced overall survival compared to those with low CD47 gene expression (Figure 4D). These findings underscore the CD47 gene's role as a significant high-risk factor influencing the prognosis of glioma patients.

The CD47 protein binds to macrophage surfaces via the SIRPα receptor, transmitting a "don't eat me" signal that inhibits macrophage phagocytosis of tumor cells, enabling immune evasion. Additionally, CD47 can activate angiogenic factors such as VEGF, promoting tumor blood vessel formation, which supplies essential nutrients and oxygen for tumor growth and metastasis^32^. Our Western blot experiments revealed that the expression level of CD47 protein in U87 and U251 cells was higher than in the normal control cell HA1800 (Figure 4E-F). Further experiments on HIF-1α's role in regulating CD47 expression in GBM by suppressing HIF-1α expression under hypoxic conditions demonstrated that compared to the shRNA-NC group, the expression of HIF-1α in U87 and U251 cells within the shRNA-HIF-1α group was significantly reduced (Figure 4G-H). Likewise, the expression levels of CD47 protein in U87 and U251 cells were found to decrease in the shRNA-HIF-1α group (Figure 4G and I). These results indicate that HIF-1α can increase the expression level of CD47 protein in GBM.

### Upregulation of CD47 protein expression by HIF-1α promotes malignant proliferation, clonal formation, and migration in GBM

**Figure 5:**
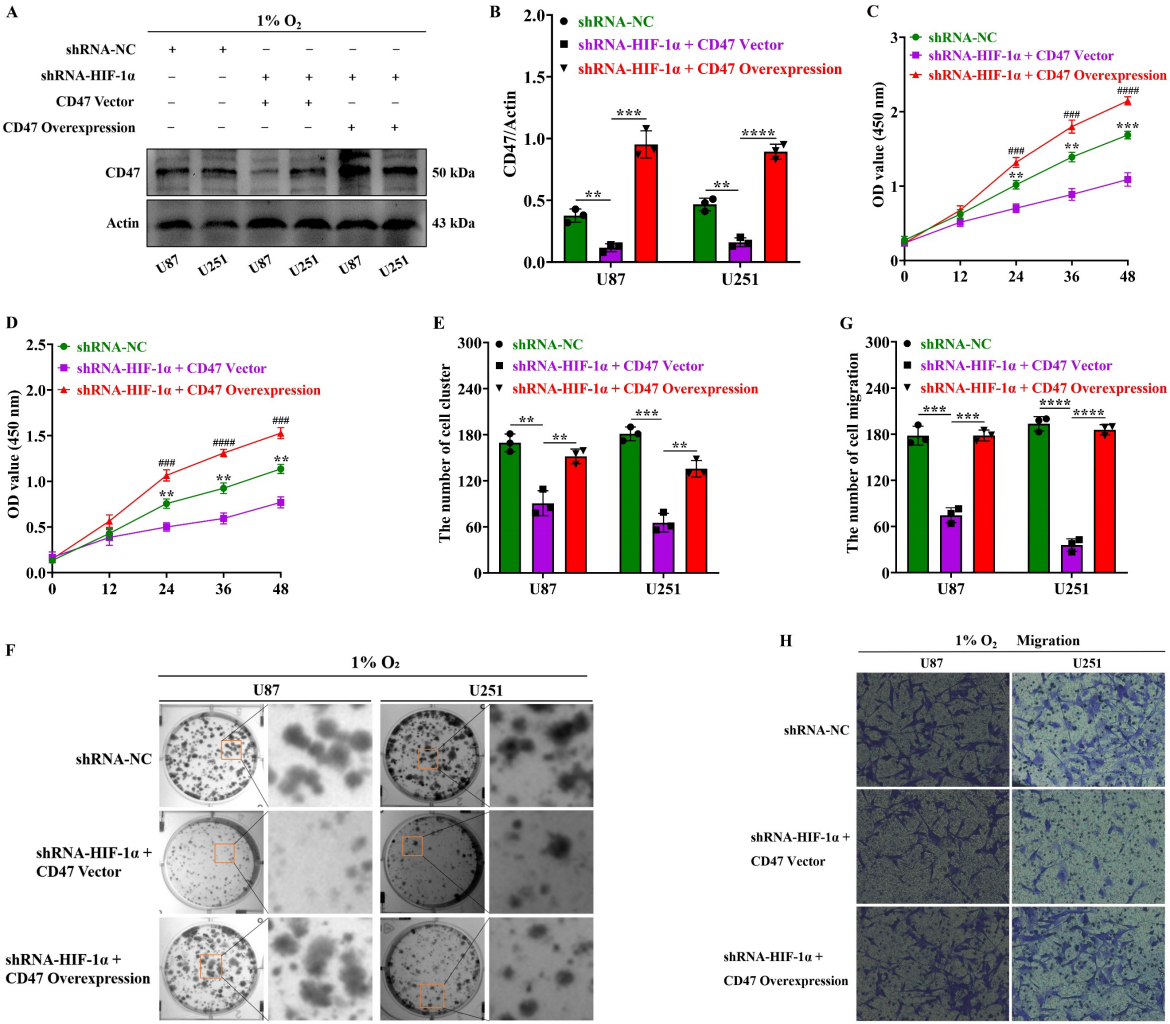
** Upregulation of CD47 promotes malignant traits in GBM via HIF-1α.** (A) Western blot showing CD47 protein expression in GBM; (B) Quantification of CD47 protein expression levels; (C) CCK8 assay assessing U87 cell proliferation after HIF-1α knockdown and CD47 overexpression; (D) CCK8 assay evaluating U251 cell proliferation after HIF-1α knockdown and CD47 overexpression. (E) Quantification of U87 and U251 cell colony formation; (F) Clonogenic assay measuring colony formation after HIF-1α knockdown and CD47 overexpression; (G) Cell migration assay assessing U87 and U251 cell migration after HIF-1α knockdown and CD47 overexpression; (H) Quantification of U87 and U251 cell migration. Data are presented as mean ± standard deviation, and differences were analyzed using an unpaired t-test. NS indicates no statistical significance, **P < 0.01, ***P < 0.001, ###*P* < 0.001, ####*P* < 0.0001.* indicates comparison between shRNA-HIF-1α + CD47 Vector and shRNA-NC; # indicates comparison between shRNA-HIF-1α + CD47 overexpression group and shRNA-HIF-1α + CD47 Vector group.

To investigate how HIF-1α modulates the malignant phenotype of GBM through CD47 protein, we transfected CD47 overexpression plasmids into GBM cells. Our findings under hypoxic conditions demonstrated that, compared to the shRNA-NC group, the expression of CD47 protein in U87 and U251 cells within the shRNA-HIF-1α group was significantly reduced. However, transfection with the CD47 overexpression plasmid effectively reversed the decrease in CD47 protein expression in the HIF-1α knockdown group (Figure 5A-B). Furthermore, to determine the function of the HIF-1α protein in regulating CD47 in GBM, we initially used the CCK8 assay to assess GBM cell proliferation and found that under hypoxic conditions, the proliferation capacity of U87 and U251 cells in the shRNA-NC group exceeded that of the shRNA-HIF-1α + CD47 plasmid control group (CD47 Vector) after 24 hours of culture. Moreover, compared to the shRNA-HIF-1α + CD47 Vector group, the proliferation ability of U87 and U251 cells in the shRNA-HIF-1α + CD47 overexpression plasmid (CD47 overexpression) group was notably higher after 24 hours of culture (Figure 5C-D). These findings suggest that the HIF-1α protein can modulate CD47 protein to enhance the proliferation capacity of GBM cells.

To further investigate how HIF-1α modulates CD47 protein in GBM, colony formation assays were conducted, and our findings under hypoxic conditions after 24 hours of culture indicated that the clonal capacity of U87 and U251 cells in the shRNA-NC group surpassed that of the shRNA-HIF-1α + CD47 Vector group. Moreover, when compared to the shRNA-HIF-1α + CD47 Vector group, the clonal ability of U87 and U251 cells in the shRNA-HIF-1α + CD47 overexpression group was significantly higher after 24 hours of culture (Figure 5E-F). These observations suggest that HIF-1α protein can regulate CD47 protein to enhance the clonal capacity of GBM cells. We further probed the role of HIF-1α in regulating CD47 protein in glioblastoma using a migration assay. Our results, under hypoxic conditions after 24 hours of culture, indicated that the migration ability of U87 and U251 cells in the shRNA-NC group exceeded that of the shRNA-HIF-1α + CD47 Vector group. Furthermore, compared to the shRNA-HIF-1α + CD47 Vector group, the migration ability of U87 and U251 cells in the shRNA-HIF-1α + CD47 overexpression group was significantly higher after 24 hours of culture (Figure 5G-H). These findings indicate that the HIF-1α protein can regulate CD47 protein to enhance the proliferation, clonal formation, and migration abilities of GBM cells.

### Knockdown of HIF-1α expression in GBM significantly inhibits the growth of subcutaneous tumors

**Figure 6:**
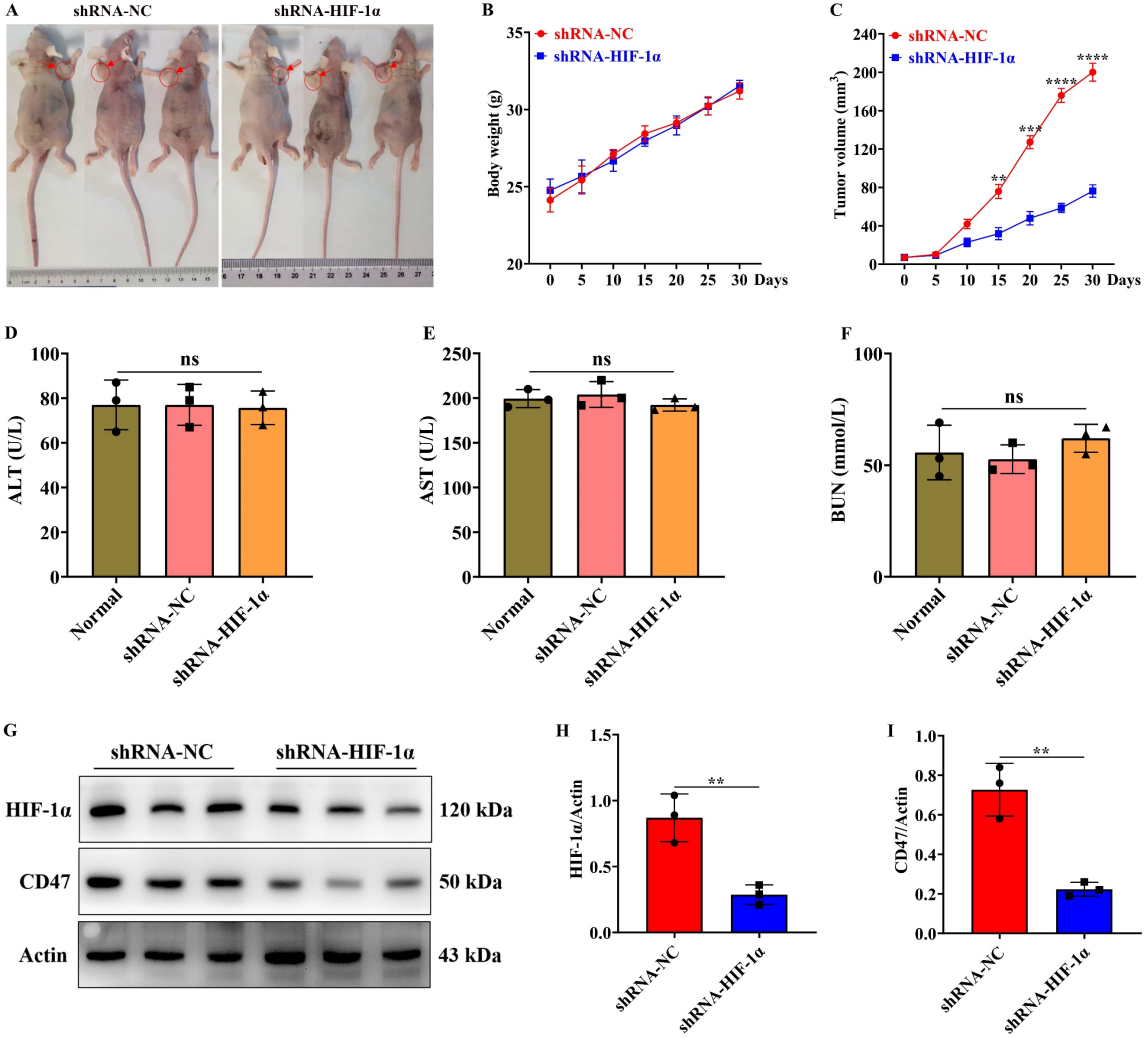
** Knockdown of HIF-1α expression inhibits subcutaneous tumor formation.** (A) Imaging of subcutaneous tumors in nude mice; (B) Changes in body weight of nude mice after subcutaneous tumor formation; (C) Changes in subcutaneous tumor volume; (D) Detection of ALT content in serum of nude mice after subcutaneous tumor formation; (E) Detection of AST content in serum of nude mice after subcutaneous tumor formation; (F) Detection of BUN content in serum of nude mice after subcutaneous tumor formation; (G) Immunoblotting showing HIF-1α and CD47 protein expression levels in subcutaneous tumor tissue; (H) Quantification of HIF-1α protein expression level; (I) Quantification of CD47 protein expression level. Data are presented as mean ± standard deviation, and differences between the two groups were analyzed using an unpaired t-test. **P <0.01, ***P <0.001, ****P <0.0001.

We further examined the impact of HIF-1α on subcutaneous tumor formation in GBM through *in vivo* experiments. Following the successful establishment of subcutaneous tumors in nude mice, we monitored the mice's weight changes every 5 days and measured the volume of the subcutaneous tumors. The experimental findings revealed no statistically significant difference in body weight changes between the shRNA-HIF-1α and shRNA-NC groups (Figure 6A-B). However, after 15 days of subcutaneous tumor formation in the shRNA-NC group, the volume of subcutaneous tumors in nude mice was notably smaller than that in the shRNA-HIF-1α group (Figure 6A and C). Furthermore, after one month of tumor formation in the nude mice, we collected serum from them and conducted tests for alanine aminotransferase (ALT), aspartate aminotransferase (AST), and blood urea nitrogen (BUN) concentrations using liver and kidney function detection kits. ALT and AST are indicators that rise in the bloodstream when the liver is damaged or hepatocytes are necrotic, reflecting the degree of liver damage. BUN concentration can provide insight into the extent of damage to glomerular filtration function and is a commonly used indicator of renal function^33^. Our research results indicated that compared to the shRNA-NC group, there were no statistically significant differences in the concentrations of ALT, AST and BUN in the serum of the shRNA-HIF-1α group (Figure 6D-F).

The experimental findings demonstrated that the protein expression level of HIF-1α was higher in the shRNA-NC group compared to the shRNA-HIF-1α group (Figure 6G-H). Similarly, we observed that compared to the shRNA-NC group, the expression level of CD47 protein in the tumor of the shRNA-HIF-1α group also decreased (Figure 6G and I). These results suggest that the knockdown of HIF-1α expression can inhibit the growth rate of GBM *in vivo* without adversely affecting the liver and kidney function of nude mice.

### Knockdown of HIF-1α expression can inhibit the P-PI3K/P-AKT, SOX2/OCT4 and MMP2/MMP9 signaling pathway *in vivo*

**Figure 7:**
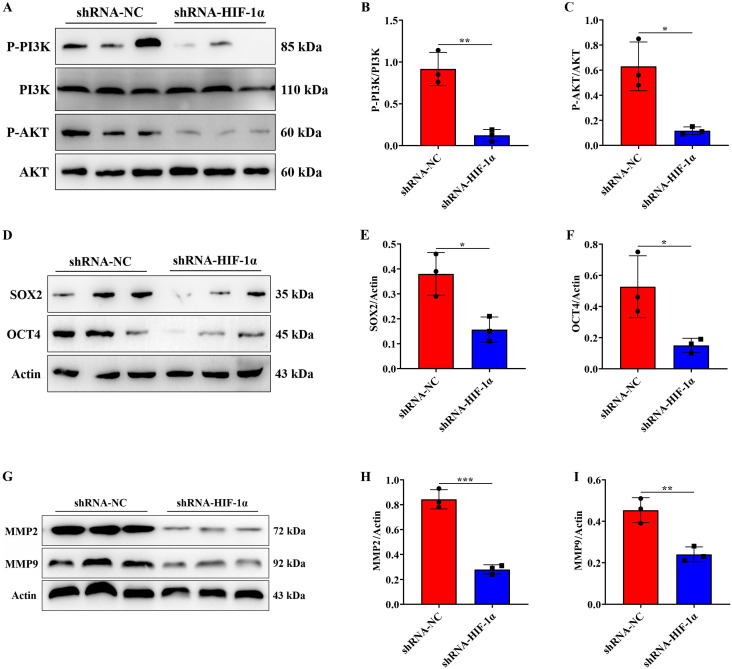
** Knockdown of HIF-1α expression can inhibit the P-PI3K/P-AKT, SOX2/OCT4 and MMP2/MMP9 signaling pathway *in vivo*.** (A) Immunoblotting showing P-PI3K, PI3K, P-AKT, and AKT protein expression levels in tumor tissue; (B) Quantification of P-PI3K protein expression level; (C) Quantification of P-AKT protein expression level; (D) Immunoblotting showing SOX2/OCT4 protein expression levels in tumor tissue; (E) Quantification of SOX2 protein expression level; (F) Quantification of OCT4 protein expression level. (G) Immunoblotting showing MMP2/MMP9 protein expression levels in tumor tissue; (H) Quantification of MMP2 protein expression level; (I) Quantification of MMP9 protein expression level. Data are presented as mean ± standard deviation, and differences between the two groups were analyzed using an unpaired t-test. *P <0.05, **P <0.01, ***P <0.001.

To elucidate the signaling pathways regulated by HIF-1α *in vivo*, we conducted further analysis to examine the expression changes of pathway proteins in tumor tissues using Western blotting and found that compared to the shRNA-NC group, the expression level of P-PI3K/P-AKT protein in the tumor of the shRNA-HIF-1α group decreased (Figure 7A-C), while the expression level of PI3K/AKT protein did not significantly change. Additionally, assessment of the expression levels of SOX2/OCT4 proteins in the tumor indicated that, compared with the shRNA-NC group, the expression level of SOX2/OCT4 proteins in the tumor of the shRNA-HIF-1α group decreased (Figure 7D-F). Furthermore, we examined the expression of MMP2/MMP9 in *in vivo* experiments, which showed that the expression levels of MMP2/MMP9 proteins in the tumor of the shRNA-HIF-1α group were decreased compared to the shRNA-NC group (Figure 7G-I). These results collectively suggest that HIF-1α can promote the malignant progression of GBM by activating the P-PI3K/P-AKT, SOX2/OCT4, and MMP2/MMP9 signaling pathways both *in vivo* and *in vitro*.

## Discussion

GBM is a highly malignant primary brain tumor characterized by a poor prognosis and limited treatment options. Despite advancements in neuro-oncology, our understanding of its underlying mechanisms remains incomplete. GBM is marked by genetic alterations, including EGFR amplification, PDGFRA mutations and IDH1/2 mutations, which drive tumor development and contribute to treatment resistance. The intricate tumor microenvironment, rich in immune cells and stromal components, also plays a pivotal role in GBM progression. Novel therapeutic approaches targeting these genetic abnormalities and the tumor microenvironment are urgently required to improve the clinical outcomes of GBM patients^34^-^36^.

HIF-1α, a pivotal transcription factor, plays a critical role in the pathogenesis of GBM^37^. GBM is characterized by its hypoxic microenvironment, leading to the stabilization and activation of HIF-1α. Activated HIF-1α contributes to tumor growth, angiogenesis, and resistance to therapy by elevating the expression of genes involved in glycolysis, cell survival, and DNA repair^38^, ^39^. In addition, HIF-1α may also interact with other oncogenic pathways, such as PI3K/AKT/mTOR and RAS/MAPK, thereby amplifying tumor progression^40^-^42^. Our analysis through the GEPIA database highlights the substantial expression of HIF-1α in GBM. Western blot detection under hypoxic conditions distinctly reveals an increase in HIF-1α protein expression in glioblastoma, emphasizing the pivotal pathogenic role played by the HIF-1α protein in GBM.

We further knocked down HIF-1α protein expression in GBM cells, resulting in a significant reduction in the proliferation, clonogenicity and migration capabilities of these cells. Multiple signal transduction pathways are involved in the proliferation, clonogenicity and migration of tumor cells via the activation of signaling pathways, including PI3K/AKT/mTOR, RAS/MAPK and Wnt/β-catenin, among others. Moreover, the tumor microenvironment plays a pivotal role in regulating cell behavior^43^. Factors such as the surrounding ECM, secreted factors and interactions with neighboring cells influence cell proliferation, migration and invasion. For instance, the secretion of transforming growth factor-β (TGF-β) by stromal cells can trigger epithelial-mesenchymal transition (EMT), a process associated with cell migration and invasion. Thus, targeting key regulators of these pathways has emerged as a promising therapeutic approach in cancer treatment. Small molecules or antibodies that inhibit kinases or other components within these pathways have demonstrated efficacy in preclinical investigations, with some receiving clinical approval. Notably, inhibitors targeting the EGFR pathway, such as gefitinib and erlotinib, have been employed in treating lung cancer patients harboring EGFR-activating mutations^44^, ^45^.

In this study, we knocked down HIF-1α protein expression in GBM cells and detected changes in associated pathway proteins by WB experiments, which showed that after knocking down HIF-1α protein expression, the expression levels of the P-PI3K/P-AKT pathway, MMPs protein family pathway and SOX2/OCT4 pathway were significantly reduced. The P-PI3K/P-AKT signaling pathway is a critical regulator of cellular processes, including proliferation, survival and metabolism. Dysregulation of this pathway has been implicated in various diseases, including cancer. The pathway is activated by various stimuli, such as growth factors and cytokines, leading to the recruitment and activation of PI3K and AKT. Activated PI3K phosphorylates phosphatidylinositol (4,5)-bisphosphate (PIP2) to generate phosphatidylinositol (3,4,5)-trisphosphate (PIP3), which recruits AKT to the plasma membrane. Subsequently, AKT is phosphorylated and activated, leading to its translocation to the nucleus, where it regulates gene expression^46^, ^47^. SOX2 and OCT4 are vital transcription factors that play pivotal roles in the self-renewal and differentiation processes of tumor cells. Their aberrant increase in expression levels has been observed in numerous tumor cells and has been associated with malignant tumor growth. The regulatory influence exerted by HIF-1α on the SOX2/OCT4 signaling pathway plays a significant role in tumor cell self-renewal and differentiation. Under hypoxic conditions, HIF-1α can stimulate the expression of SOX2 and OCT4, thereby facilitating tumor cell self-renewal and differentiation processes. Additionally, SOX2 and OCT4 can synergize with HIF-1α to promote tumor cell migration and invasion^48^-^50^.

MMP2 and MMP9 mediate important steps in tumor angiogenesis by degrading ECM and basement membrane (BM), creating a conducive environment for the generation and extension of new blood vessels. Consequently, endothelial cells can detach from their original vascular structures, facilitating the migration of tumor cells along the basement membrane and inciting the process of angiogenesis. The proteolytic functions of MMP2 and MMP9 enable tumor cells to breach vascular walls and gain access to the bloodstream, ultimately culminating in distant metastasis^51^, ^52^.

We further discovered that HIF-1α could upregulate the expression of CD47 protein in GBM, suggesting that HIF-1α may influence CD47 in GBM's malignant progression. Our experiments confirmed that reducing HIF-1α while increasing CD47 in GBM cells significantly enhances malignant proliferation, colony formation and tumor cell migration, indicating that HIF-1α can modulate GBM characteristics via the CD47 protein. The precise mechanisms involving CD47 in tumor development and progression remain incompletely understood. Recent studies reveal that CD47 can interact with its receptor, signal-regulatory protein alpha (SIRPα), on phagocytic cells like macrophages and dendritic cells to inhibit their ability to engulf cancer cells, promoting immune evasion and tumor growth. Additionally, CD47 can interact with other proteins, such as integrins and selectins, impacting cell adhesion and migration. Consequently, targeting CD47 holds promise in enhancing the effectiveness of anti-cancer therapies by restoring the immune system's capacity to recognize and eliminate cancer cells^53^, ^54^.

Nonetheless, several limitations exist in this study. First, we have yet to elucidate the precise mechanism through which HIF-1α regulates the overexpression of CD47 protein in GBM. Second, while investigating these regulatory mechanisms, we did not simultaneously knock down HIF-1α and restore CD47 protein expression in GBM cells to investigate alterations in pathway proteins. Finally, our *in vivo* experiments did not assess the roles of HIF-1α and CD47 protein in GBM's tumorigenesis and underlying mechanisms.

## Figures and Tables

**Table 1:** The sequences of primers used in this experiment.

Gene name	Sequences of primers
Actin	Forward: 5'-AGTTGCGTTACACCCTTTCTTG-3'; Reverse: 5'-GCTGTCACCTTCACCGTTCC-3'.
HIF-1α	Forward:5'-AGCCTGTTCACCTATGTTG-3'; Reverse: 5'-CCTTGTCCATGAGGTTGTA-3'.

**Table 2:** The sequences of shRNA used in this experiment.

Gene name	Sequences of primers
shRNA-NC	5'-TCTCGCAGCCATTGTCAC-3'
shRNA-HIF-1α	5'-GATCCGTTGTCTGGATGTT-3'
